# SsAGM1-Mediated Uridine Diphosphate-*N*-Acetylglucosamine Synthesis Is Essential for Development, Stress Response, and Pathogenicity of *Sclerotinia sclerotiorum*

**DOI:** 10.3389/fmicb.2022.938784

**Published:** 2022-06-23

**Authors:** Junting Zhang, Kunqin Xiao, Maoxiang Li, Hanlong Hu, Xianghui Zhang, Jinliang Liu, Hongyu Pan, Yanhua Zhang

**Affiliations:** College of Plant Sciences, Jilin University, Changchun, China

**Keywords:** *Sclerotinia sclerotiorum*, pathogenicity, SsAGM1, infection cushion, sclerotia, chitin, UDP-GlcNAc

## Abstract

The necrotrophic fungus *Sclerotinia sclerotiorum* is a devastating pathogen. *S. sclerotiorum* can cause Sclerotinia stem rot in more than 600 species of plants, which results in serious economic losses every year. Chitin is one of the most important polysaccharides in fungal cell walls. Chitin and β-Glucan form a scaffold that wraps around the cell and determines the vegetative growth and pathogenicity of pathogens. UDP-GlcNAc is a direct precursor of chitin synthesis. During the synthesis of UDP-GlcNAc, the conversion of GlcNAc-6P to GlcNAc-1P that is catalyzed by AGM1 (*N*-acetylglucosamine-phosphate mutase) is a key step. However, the significance and role of AGM1 in phytopathogenic fungus are unclear. We identified a cytoplasm-localized SsAGM1 in *S. sclerotiorum*, which is homologous to AGM1 of *Saccharomyces cerevisiae*. We utilized RNA interference (RNAi) and overexpression to characterize the function of *SsAGM1* in *S. sclerotiorum*. After reducing the expression of *SsAGM1*, the contents of chitin and UDP-GlcNAc decreased significantly. Concomitantly, the gene-silenced transformants of *SsAGM1* slowed vegetative growth and, importantly, lost the ability to produce sclerotia and infection cushion; it also lost virulence, even on wounded leaves. In addition, *SsAGM1* was also involved in the response to osmotic stress and inhibitors of cell wall synthesis. Our results revealed the function of *SsAGM1* in the growth, development, stress response, and pathogenicity in *S. sclerotiorum*.

## Introduction

The ascomycete *Sclerotinia sclerotiorum* (Lib.) de Bary is an important necrotrophic, soilborne, phytopathogenic fungus, which is distributed throughout the world (Boland and Hall, 1994). *S. sclerotiorum* is remarkable for its extremely broad host range, and diseases caused by this pathogen occur in more than 600 plant species, which include almost all dicotyledons and some monocotyledons (Liang and Rollins, 2018; Xia et al., 2019). It can infect many crops of economic importance, such as oil crops including soybeans, *Brassica napus*, and sunflowers (Boland and Hall, 1994); vegetable crops including lettuce and tomatoes, and ornamental crops including tulips and chrysanthemums (Bolton et al., 2006).

Importantly, the damage caused by *S. sclerotiorum* is enormous globally every year (Xu T. et al., 2018). The vegetative hyphae of *S. sclerotiorum* interweave and gather together to formed hard, black sclerotia (Erental et al., 2008). Sclerotia is the resting and propagation structure of *S. sclerotiorum*, which can survive in the soil for many years where it resists adverse environmental conditions (Zhang et al., 2021; Yu and Rollins, 2022). In addition, *S. sclerotiorum* has a massive and complex pathogenetic arsenal, which includes an array of cell wall–degrading enzymes, phytotoxins, and other secondary metabolites that kill host cells and facilitate infection (Kabbage et al., 2013; Seifbarghi et al., 2017; Xu L. et al., 2018; Allan et al., 2019). *S. sclerotiorum* can also secrete oxalic acid to inhibit host immune responses in the early stage of infection, and it induces cell necrosis in the later stage of infection (Kim et al., 2008; Williams et al., 2011). Recent investigations showed that there is a very complex mechanism of interaction between *S. sclerotiorum* and its hosts (Mbengue et al., 2016; Liang and Rollins, 2018). It is extremely difficult to control the disease caused by *S. sclerotiorum* due to its extensive host range, complex pathogenesis, and the strong survival ability of sclerotia. At present, chemical control is used mainly to control Sclerotinia diseases because there is a lack of resistant cultivars (Seifbarghi et al., 2017; Xia et al., 2019). Therefore, identifying and characterizing the growth, development, and pathogenesis are helpful to explore potential drug targets and to provide a new strategy for controlling diseases caused by *S. sclerotiorum*.

The fungal cell wall is a rigid but dynamic structure, which is essential for the growth, development, and pathogenicity of plant pathogenic fungi (Gow et al., 2017; Patel and Free, 2019; Plaza et al., 2020). Furthermore, a series of elegant studies has shown recently that cell walls have extraordinary effects on many aspects of fungal physiology, which include cell survival, morphogenesis, virulence, perception of environmental changes, and host-pathogen interactions (Geoghegan et al., 2017; Latge et al., 2017; Alcazar-Fuoli et al., 2020). In *Saccharomyces cerevisiae*, one-fifth of the yeast genome, or about 1,200 genes, is devoted to the biosynthesis of the cell wall, which includes the assembly of basic components, the supply of substrates, signal transduction, and regulation (de Groot et al., 2001; Klis et al., 2006). This fact also demonstrates the importance of fungal cell walls. The cell wall of fungi is composed mainly of chitin, mannan, and glucan, and polysaccharides account for about 90% of the cell wall (Klis et al., 2006; Oliveira-Garcia and Deising, 2013). Chitin accounts for 10–20% of the total cell wall contents of filamentous fungi (Klis, 1994; Arroyo et al., 2016). Chitin plays an essential role in maintaining cell wall integrity and pathogenicity of phytopathogenic fungi (Arroyo et al., 2016; Patel and Free, 2019). Therefore, many enzymes and regulatory factors in the process of chitin biosynthesis can be used as effective targets of fungicides.

The biosynthesis of chitin in fungi initiates from glucose, mannose, and fructose, and which are transformed into Fructose 6-phosphate (Fru-6P) through a series of biochemical reactions (Hofmann et al., 1994). Fru-6P is converted to glucosamine 6-phosphate by aminoamide: fructose-6-phosphate aminotransferase (GFA1). Glucosamine 6-phosphate is further *N*-acetylated by GlcN-6P acetyltransferase (GNA1) to form GlcNAc-6P (Fang et al., 2013). GlcNAc-6P is converted to GlcNAc-1P by *N*- acetylglucosamine-phosphate mutase (AGM1), which catalyzes intramolecular phosphoryl transfer on arrange of phosphosugar substrates (Milewski et al., 2006; Fang et al., 2013). GlcNAc-1P is transformed into UDP-GlcNAc (uridine diphosphate-*N*-acetylglucosamine) under the action of UDP-GlcNAc pyrophosphorylase (UAP1) (Milewski et al., 2006). UDP-GlcNAc is the direct precursor of chitin (Free, 2013). Finally, chitin is synthesized by plasma membrane-associated chitin synthases (Free, 2013; Gow et al., 2017). In this pathway, the conversion of GlcNAc-6P to GlcNAc-1P that is catalyzed by AGM1 is a vital step in the synthesis of UDP-GlcNAc.

AGM1 has been isolated and identified from *S. cerevisiae*, *Aspergillus fumigatus*, *Candida albicans*, and *Homo sapiens* (Hofmann et al., 1994; Mio et al., 2000; Fang et al., 2013). In yeast, *AGM1* is an essential gene for cell survival. The deletion of *AGM1* gene resulted in abnormal division and a decrease in survival rate (Hofmann et al., 1994). Mice that lacked the *AGM1* homologous gene (*pgm3*) died before implantation, and heterozygotes had congenital hematopoietic and reproductive defects (Greig et al., 2007). In the human pathogenic fungus *A. fumigatus*, reducing the expression of *AfAGM1* resulted in defects in hyphae growth, and melanin synthesis (Fang et al., 2013). However, the function of AGM1 in phytopathogenic fungi is poorly understood.

In this study, we identified *SsAGM1* (i.e., the homolog of *AGM1* in *S. cerevisiae*) in *S. sclerotiorum*. We employed RNA interference (RNAi) and overexpression to characterize the function of *SsAGM1* in *S. sclerotiorum*. We showed that UDP-GlcNAc synthesis that was mediated by SsAGM1 affected many aspects of *S. sclerotiorum*, which included the content of chitin in the cell wall, cell wall integrity, mycelial growth, formation of infection cushions, sclerotia formation, stress response, and pathogenicity. We demonstrated that *SsAGM1* was essential for the growth, development, and pathogenicity of *S. sclerotiorum*.

## Materials and Methods

### Fungal Strains and Culture Conditions

In this study, *S. sclerotiorum* strain UF-1, which was isolated from infected petunias in Florida, United States, was used as a wild-type strain (WT) (Li et al., 2018). WT and all transgenic strains in this study were initially grown on potato glucose agar (PDA) plate (200 g potato, 20 g glucose, 15 g agar, constant volume to 1 L) and cultured at 25°C in the dark. *Arabidopsis thaliana* (*Arabidopsis* ecotype Col-0), *Glycine max* (soybean Williams 82), and *Solanum lycopersicum* (tomato Zhongshu 4) were grown in the glasshouse under natural sunlight with temperature in the 22–25°C range.

### Identification and Sequence Analysis of *SsAMG1*

The gene and protein sequences of *SsAMG1* were obtained from the NCBI database,^1^ and the conserved domain of SsAMG1 was predicted by BLAST and the conserved domain database. The homologous protein of SsAMG1 in other species was obtained from NCBI and related literature. Multiple sequence alignments were constructed with ClustalW with full-length protein sequences (Thompson et al., 1994). A phylogenetic tree was constructed using MEGA 5 with the neighbor-joining algorithm under default settings and 1,000 bootstrap replications (Tamura et al., 2007).

### Plasmid Constructs and Transformation

The pSilent-Dual1 and pYF11 (Fan et al., 2017; Jiao et al., 2022) vectors, which were used for gene silencing and overexpression vectors in this study, respectively, contained a geneticin (G418) resistance marker. To silence *SsAGM1* of *S. sclerotiorum*, four fragments of the coding region of *SsAGM1* were amplified with specific primers that used *S. sclerotiorum* cDNA as a template. The PCR products were ligated into the *Xba*I restriction site of the pSilent-Dual1 vector to generate pSD-SsAGM1-T1, pSD-SsAGM1-T2, pSD-SsAGM1-T3, and pSD-SsAGM1-T4 constructs (Supplementary Figure 1). To generate *S. sclerotiorum* overexpression strains of *SsAGM1*, the full-length coding region was amplified with specific primers that used *S. sclerotiorum* cDNA as a template. The amplified product was connected to the pYF11 vector that was linearized with *Xho*I restriction enzyme by seamless cloning to produce the pYF11: SsAGM1:eGFP vector. The above vectors were verified by sequencing and then used for genetic transformation of *S. sclerotiorum*. The pSilent-Dual1 was used as an empty vector control. All primers used in this assay are listed with brief descriptions in Supplementary Table 1.

The preparation of *S. sclerotiorum* protoplasts was carried out as described previously (Qu et al., 2014). These constructs were transferred into *S. sclerotiorum* protoplasts by the PEG-mediated transformation protocol (Rollins, 2003). Transformants were screened three times with a PDA plate that contained G418 (100 μg/mL).

### Reverse-Transcription, Quantitative Real-Time PCR Analysis

All strains were inoculated on a PDA that was covered with cellophane at 25°C in the dark. The hyphae were collected and frozen immediately in liquid nitrogen. Total RNA was extracted and purified by a TransZol Up Plus RNA Kit (TransGen Biotech). Total RNA was reverse transcribed using EasyScript All-in-One First-Strand cDNA Synthesis SuperMix (TransGen Biotech) to synthesize cDNA that was used for subsequent experiments. cDNA was diluted 10 times as a template. Reverse-transcription, quantitative real-time PCR (RT-qPCR) was performed with the TransStart Green qPCR SuperMix (TransGen Biotech). *SsActin* of *S. sclerotiorum* was used as an internal reference. The relative expression levels were calculated with three technical replications using the 2^–ΔΔCt^ method. All primers used in this assay are listed with brief descriptions in Supplementary Table 1. All experiments were repeated three times independently.

### Measurement of the Content of Chitin and Uridine Diphosphate-*N*-Acetylglucosamine in Hyphae

The chitin content was determined according to the method described previously with modifications (Bulik et al., 2003). First, the mycelial samples were dried and ground sufficiently. For each sample, 5 mg of dried mycelium was resuspended in 1 mL 6% KOH and heated at 80°C for 90 min. After cooling to room temperature, the sample was centrifuged (1,600 × g, 10 min) and resuspended with phosphate buffered saline, and this was repeated three times. Finally, the sample was resuspended in 500 μL McIlvaine’s buffer (pH 6.0), mixed gently with 100 μL *Streptomyces duplicatus* chitinase (Sigma), and incubated at 37°C for 18 h. We extracted 100 μL from each sample, and these were mixed fully with 100 μL 0.27 M NaB_4_O_7_ (pH 9.0) were fully mixed in 2 mL centrifuge tubes and heated at 98°C for 10 min. Then, 1 mL Ehrlich’s reagent (10 g β-dimethylaminobenzaldehyde in 1.25 mL of concentrated HCl and 98.75 mL glacial acetic acid) was added to each sample and incubated at 37°C for 20 min. One milliliter of the sample was transferred to a cuvette and the absorbance at 585 nm was recorded. A standard curve was prepared with GlcNAc (BBI, China). All experiments were repeated three times independently.

To determine the content of UDP-GlcNAc in WT, control strain (CK), and in gene-silenced and overexpression transformants, the mycelia of all strains were inoculated in liquid PD medium (200 g potato, 20 g glucose per liter) for 3 days at 25°C in the dark. The method for determining the UDP-GlcNAc content was performed as described in the manual (Qingdao Kechuang, China).

### Mycelia Growth, Sclerotia, and Formation of Infection Cushions

To observe mycelia growth and sclerotia formation, the WT, CK, gene-silenced transformants, and overexpression transformants were inoculated on PDA plates for 2–15 days at 25°C in the dark; colony diameters were measured every 12 h. Infection cushions of strains were observed on glass slides. The formation of infection cushions was observed under a microscope at 12 h after inoculation. All experiments were repeated three times independently.

### Determinations of Cell Wall Sensitivity and Tolerance of Osmotic Stress

We determined the inhibition rate of hyphal growth when cultured with inhibitors of cell wall synthesis and external osmotic substances. Agar plugs with actively growing hyphae that were obtained from WT, CK, gene-silenced transformants, and overexpression transformants were inoculated on PDA plates that contained Congo red (CR, 500 μg/mL), CFW (50 μg/mL, SIGMA, 910090), 0.005% SDS, 1 M sorbitol, 0.7 M KCl, and 0.7 M NaCl. The colony diameters were measured, and the inhibition rate of hyphae growth was calculated at 48 h after inoculation. Inhibition rate = (diameter of untreated strain—diameter of treated strain)/(diameter of untreated strain × 100%). All experiments were conducted three times independently.

### Subcellular Localization of SsAGM1

To observe the subcellular localization of SsAGM1, overexpression transformants *OE-SsAGM1*-2 and *OE-SsAGM1*-11 were inoculated on PDA medium and covered with cellophane for 12 h at 25°C in the dark. The hyphae were viewed under a fluorescence microscope with a 480-nm light.

### Pathogenicity Assays

The pathogenicity of *S. sclerotiorum* was determined by infecting leaves of soybean, *Arabidopsis*, and tomato. First, the WT, CK, gene-silenced transformants, and overexpression transformants were cultured on PDA plates. Agar plugs (0.5 cm diameter) were obtained from the edge of PDA-cultured colonies and inoculated on intact soybean, *Arabidopsis*, intact tomato leaves, and wounded tomato leaves at room temperature. The inoculated leaves were kept in a sealed container with high humidity for 4 days. The lesion area was measured and photographed at 36 and 48 h after inoculation, and the lesion area was calculated using ImageJ. Pathogenicity tests were repeated three times independently.

### Statistical Analysis

Data were tested for homogeneity of variances first. If variances were equal, statistical analysis was performed with one-way ANOVA using SPSS. Pairwise comparisons were performed with Tukey’s HSD (Honestly Significant Difference) using SPSS. In this study, all data were expressed as the mean ± SD, and the *p*-value < 0.05 was considered statistically significant. Statistical charts were constructed using GraphPad Prism 8.0.2.

## Results

### Identification of *SsAGM1* in *Sclerotinia sclerotiorum*

**FIGURE 1:**
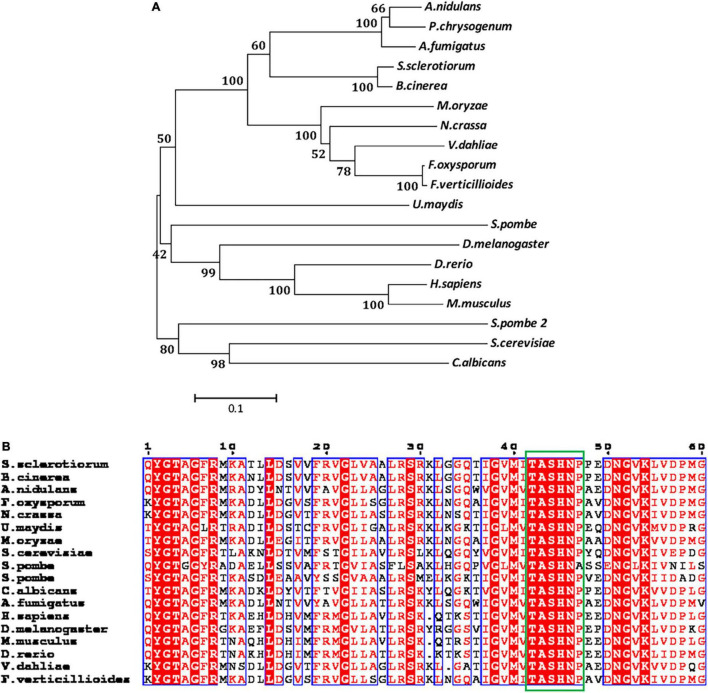
*SsAGM1* encodes an *N*-acetylglucosamine-phosphate mutase in *Sclerotinia sclerotiorum*. **(A)** Phylogenetic analysis of SsAGM1 and its homologs. The amino acid sequences of SsAGM1 and its homologs were aligned using ClustalW, and the phylogenetic tree was constructed using MEGA 5 with the neighbor-joining algorithm. GenBank accession numbers of *S. sclerotiorum* and other species are in Supplementary Table 2. **(B)** Alignments of the conserved domains in SsAGM1 and its homologs. Alignments of the conserved domains by ClustalW and shown by ESPript 3.0. The conserved sequence motif Ser/Thr-X-Ser-His-Asn-Pro is shown in the green box. Residues with a score above defined threshold are written in red and boxed in blue.

The SS1G_01582 of *S. sclerotiorum* is the ortholog of *S. cerevisiae AGM1*(YEL058W). SS1G_01582 has a 1,667-bp open reading frame, which encodes a predicted protein with a length of 538 amino acids. The ORF of the SS1G-01582 gene was interrupted by a 50-bp intron. The protein encoded by the SS1G_01582 was annotated in GenBank, which contained the conserved domain of AGM1 (*N*-acetylglucosamine-phosphate mutase). Protein BLAST searches performed with AGM1 proteins of *S. cerevisiae* and *A. fumigatus* suggested that AGM1 of *S. sclerotiorum* shared 66.61 and 45.86% sequence identity with *S. cerevisiae* AGM1 and *A. fumigatus* AfAGM1, respectively. Therefore, we designated this gene as *SsAGM1*. The phylogenetic tree analysis demonstrated that SsAGM1 sequences from *S. sclerotiorum* and *Botrytis cinerea* Bcpcm1 (Accession number: XP_024551673.1) formed a single clade, with a bootstrap of 100% (Figure 1A). The sequence motif Ser/Thr-X-Ser-His-Asn-Pro is highly conserved, and serine phosphorylation at the third position is necessary to give full play to its activity (Raimi et al., 2018). Through the alignment of the amino acid sequence of the conserved domain, we found that the conserved sequence motif Ser/Thr-X-Ser-His-Asn-Pro was contained in SsAGM1 (Figure 1B).

**FIGURE 2:**
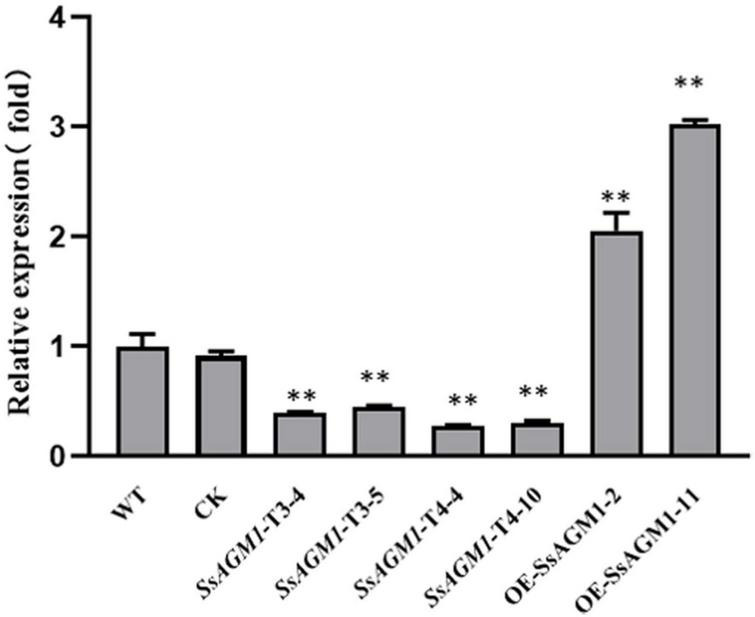
The relative expression level of *SsAGM1* in wild-type strain, control strain, gene-silenced transformants, and overexpression transformants was measured by RT-qPCR. The *SsActin* in each sample was used as an internal control. The relative expression of *SsAGM1* in WT was set as “1.” Data represent means ± SD. One-way ANOVA, *n* = 3; ^**^ represents significant difference from WT at *p* < 0.05. WT, Wild-type strain; CK, the control strain.

To elucidate the function of *SsAGM1* in *S. sclerotiorum*, we replaced *SsAGM1* with *hyg* by homologous recombination to obtain a knockout mutant. PCR screens performed with many transformants always led to amplification of an 800 bp fragment of the *SsAGM1*, which indicated that *SsAGM1* was not replaced completely. In *S. cerevisiae* and *A. fumigatus*, the *AGM1* gene was lethal (Hofmann et al., 1994; Fang et al., 2013). We speculated that *SsAGM1* was also a lethal gene in *S. sclerotiorum*. Therefore, we employed RNA interference and obtained the gene-silenced transformants of *SsAGM1*. The transformants obtained from the pSilent-Dual1 empty vector was used as the control strain (CK) in this study. The expression of *SsAGM1* in gene-silenced transformants *SsAGM1*-T3-4, *SsAGM1*-T3-5, *SsAGM1*-T4-4, and *SsAGM1*-T4-10 decreased by about 60–70% compared with WT and CK (*SsAGM1*-T3-4: decreased by 60%, *SsAGM1*-T3-5: decreased by 55%, *SsAGM1*-T4-4: decreased by 72%, *SsAGM1*-T4-10 decreased by 70%) (Figure 2). There was no obvious difference in the expression of *SsAGM1* between WT and CK.

In addition, we constructed the gene-overexpression vector pYF11:SsAGM1:eGFP, and the overexpression transformants of *SsAGM1* were obtained. The expression of *SsAGM1* in *OE- SsAGM1*-2 and *OE-SsAGM1*-11 was up-regulated by 2–3 times compared with WT (Figure 2). Furthermore, fluorescence microscopy showed that SsAGM1- GFP fusion proteins were distributed in the cytoplasm of *S. sclerotiorum* (Supplementary Figure 2). These data indicated that we identified a conserved SsAGM1 that was essential for the survival of *S. sclerotiorum*, and it was located in the cytoplasm.

### *SsAGM1* Was Involved in Uridine Diphosphate-*N*-Acetylglucosamine and Chitin Synthesis in *Sclerotinia sclerotiorum*

**FIGURE 3:**
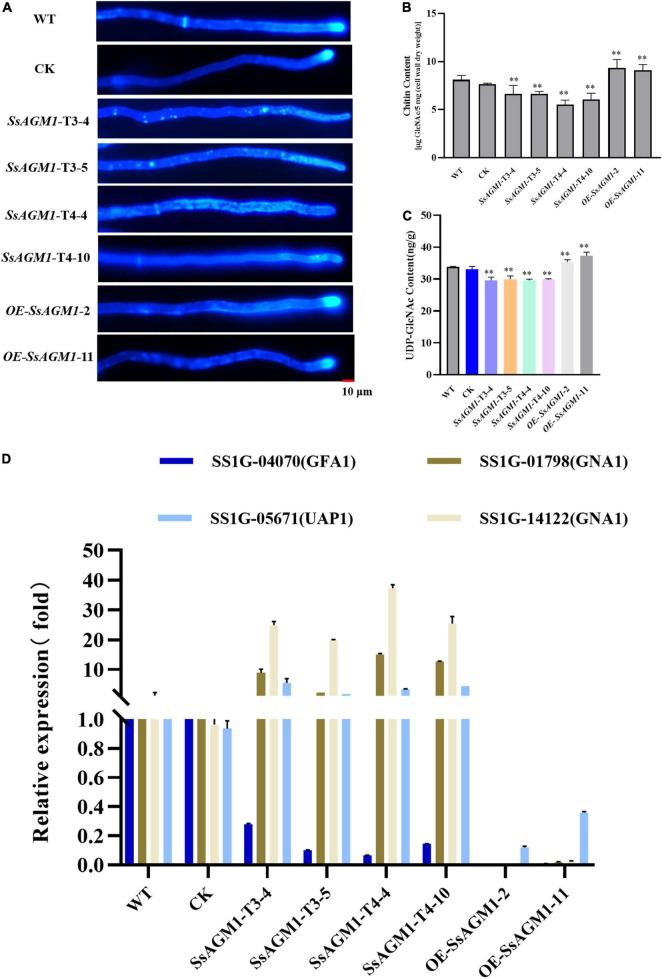
*SsAGM1* is involved in UDP-GlcNAc and chitin synthesis in *Sclerotinia sclerotiorum*. **(A)** Wild-type strain, control strain, gene-silenced transformants, and overexpression transformants were treated with Calcofluor white (CFW) and observed using a fluorescence microscope. Strains were inoculated on PD for 12 h, and hyphae were stained by CFW (10 μg/mL) for 3 min in the dark. Scale bars, 10 μm. WT, Wild-type strain; CK, the control strain. **(B)** The determination of chitin content used the fluorimetric Morgan-Elson method. **(C)** Quantitation of UDP- GlcNAc content. Data represent means ± SD, *n* = 3. One-way ANOVA; ^**^represents significant difference from WT at *p* < 0.05. **(D)** The analysis of the expression of key genes in the UDP-GlcNAc synthesis pathway by RT-qPCR. GFA1, glutamine: Fru-6P amidotransferase; GNA1, GlcN-6P acetyltransferase; UAP1, UDP-GlcNAc pyrophosphorylase. Error bars: the standard deviation of three replicates.

Calcoflour white (CFW) is a common fluorochrome used to detect defective mutants in the cell wall (Ram and Klis, 2006). To clarify the function of *SsAGM1* in chitin synthesis, we stained the hyphae of the WT, CK, gene-silenced transformants, and overexpression transformants with CFW. In WT, CK, and overexpressed transformants, fluorescence gathered mainly at the septa and tips of hyphae, which are the most active places of chitin synthesis. However, the fluorescence was not concentrated at the hyphal tips in gene-silenced transformants, but it was distributed randomly and exhibited an irregular dot aggregation in the hyphae of gene-silenced transformants (Figure 3A). CFW staining showed that chitin synthesis and distribution were abnormal. AGM1 is required for UDP-GlcNAc synthesis (Milewski et al., 2006). We analyzed the accumulation of UDP-GlcNAc in hyphae quantitatively. The content of UDP-GlcNAc decreased by about 15% in *SsAGM1* gene-silenced transformants compared with WT and CK, whereas the content of UDP-GlcNAc increased by 10% in overexpressed transformants compared with WT (Figure 3C). In addition, we also analyzed the content of chitin in the cell wall of strains quantitatively. The content of chitin decreased by about 20–30% in *SsAGM1* gene-silenced transformants compared with WT and CK, whereas the content of chitin increased by about 15% in overexpressed transformants compared with WT (Figure 3B). There was no obvious difference in the content of UDP-GlcNAc and chitin between WT and CK.

UDP-GlcNAc is synthesized from Fru-6P catalyzed successively by GFA1, GNA1, AGM1, and UAP1 (Milewski et al., 2006). We explored the effect on the expression of other genes in the UDP-GlcNAc synthesis pathway further after reducing the expression of *SsAGM1*. RT-qPCR was used to detect the expression of SS1G_04070 (GFA1), SS1G_01798 (GNA1), SS1G_14122 (GNA1), and SS1G_05671 (UAP1) of *S. sclerotiorum*. Compared with WT and CK, the expression of SS1G_05671, SS1G_01798, and SS1G_14122 increased significantly in *SsAGM1* gene-silenced transformants, and the expression of SS1G_04070 decreased significantly. The expression of four genes in the overexpressed transformants decreased significantly compared with WT (Figure 3D). There was no significant difference in the expression of four genes between CK and WT. The results demonstrated that *SsAGM1* was involved in UDP-GlcNAc and chitin synthesis in *S. sclerotiorum*.

### *SsAGM1* Contributed to Stress Tolerance in *Sclerotinia sclerotiorum*

**FIGURE 4:**
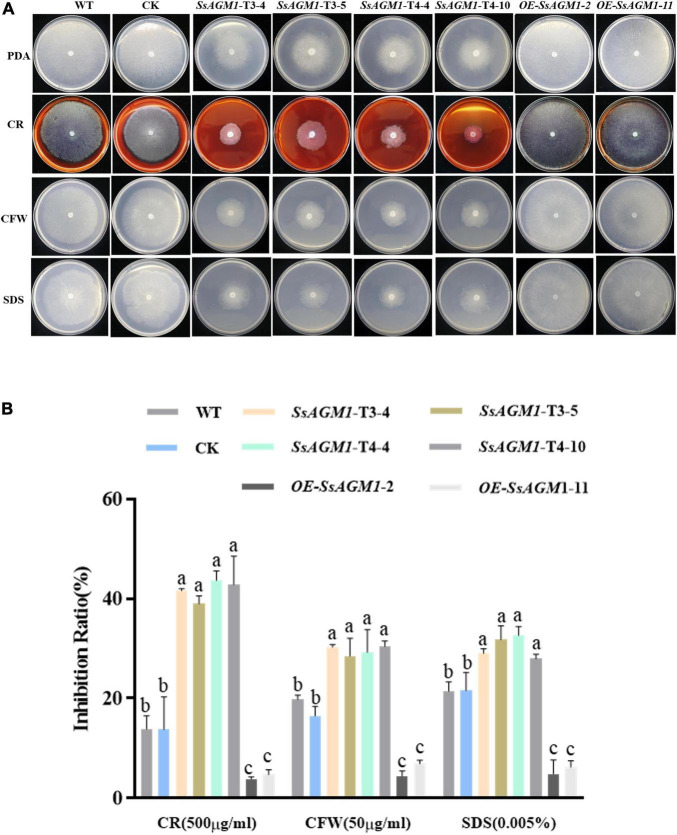
*SsAGM1* contributes to the tolerance to inhibitors of cell wall synthesis in *Sclerotinia sclerotiorum*. **(A)** The growth phenotypes of wild-type strain, control strain, gene-silenced transformants, and overexpression transformants on PDA supplemented with CR, SDS, and CFW for 48 h after inoculation. WT, Wild-type strain; CK, the control strain; CFW, Calcofluor white; CR, Congo red; SDS, sodium dodecyl sulfate. **(B)** The inhibition rate of hyphae growth on PDA supplemented with different inhibitors of cell wall synthesis. Inhibition rate = (diameter of untreated strain - diameter of treated strain)/(diameter of untreated strain × 100%). Data represent means ± SD. One-way ANOVA; *n* = 3; different letters indicate statistically significant differences (*p* < 0.05).

Because chitin is an important component of cell walls (Patel and Free, 2019), we examined the role of *SsAGM1* in the response to inhibitors of cell wall synthesis in *S. sclerotiorum*. CFW, congo red (CR), and sodium dodecyl sulfonate (SDS) are synthesis inhibitors of cell walls, and they are also common chemicals for detecting cell wall-defective mutants (Ram and Klis, 2006). Therefore, WT, CK, gene-silenced transformants, and overexpression transformants were inoculated on a PDA that contained CR, CFW, and SDS. We found that the tolerance of gene-silenced transformants to inhibitors of cell wall synthesis decreased significantly compared with WT and CK, although the tolerance to inhibitors of cell wall synthesis of overexpression transformants increased significantly compared with WT (Figures 4A,B). There was no significant difference in the tolerance to inhibitors of cell wall synthesis between CK and WT. The results suggested that *SsAGM1* regulated the tolerance to inhibitors of cell wall synthesis positively, and it may be involved in maintaining cell wall integrity in *S. sclerotiorum*.

**FIGURE 5:**
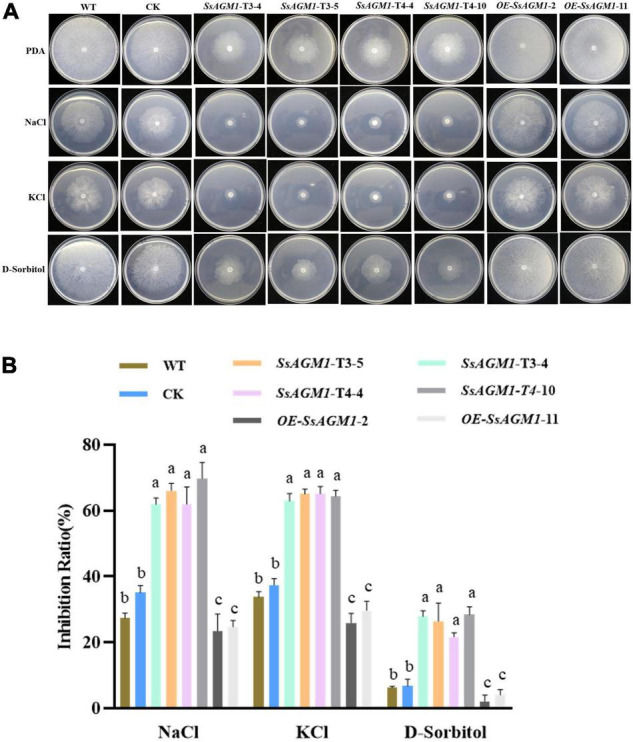
*SsAGM1* contributes to the response to osmotic stress in *Sclerotinia sclerotiorum.* **(A)** The growth phenotypes of wild-type strain, control strain, gene-silenced transformants, and overexpression transformants on PDA supplemented with NaCl, KCl, and D-sorbitol for 48 h after inoculation. **(B)** The inhibition rate of hyphae growth on PDA supplemented with different external osmotic substances. Inhibition rate = (diameter of untreated strain - diameter of treated strain)/(diameter of untreated strain × 100%). One-way ANOVA; *n* = 3; different letters indicate statistically significant differences (*p* < 0.05). Data represent means ± SD. WT, Wild-type strain; CK, the control strain.

Furthermore, we investigated whether *SsAGM1* was involved in other stresses, such as osmotic stress. The WT, CK, gene-silenced transformants, and overexpression transformants were inoculated on a medium that contained NaCl, KCl, and D-sorbitol. The growth inhibition rate of gene-silenced transformants was significantly higher than in WT or CK (Figures 5A,B). However, the growth inhibition rate of overexpression transformants was significantly lower than in WT (Figures 5A,B). There was no significant difference in the growth inhibition rate between CK and WT. This indicated that *SsAGM1* also positively regulated tolerance to osmotic stress of *S. sclerotiorum*. Thus, these results showed that *SsAGM1* contributed to a variety of stress tolerance in *S. sclerotiorum*.

### *SsAGM1* Was Involved in Vegetative Growth and Sclerotia Formation in *Sclerotinia sclerotiorum*

**FIGURE 6:**
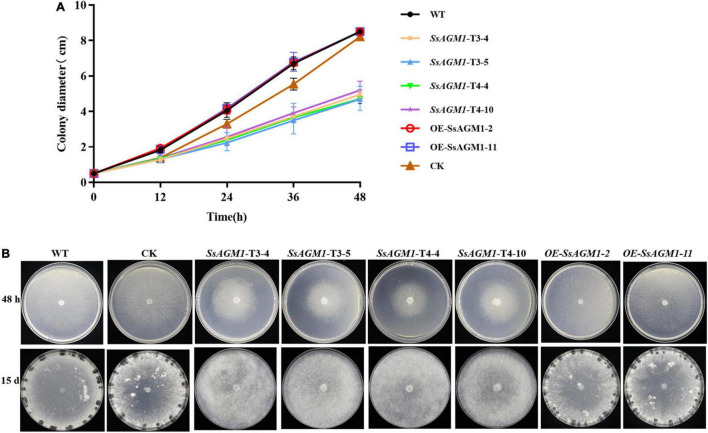
*SsAGM1* is involved in vegetative growth and sclerotia formation in *Sclerotinia sclerotiorum*. **(A)** The hyphae growth rate in wild-type strain, control strain, gene-silenced transformants, and overexpression transformants. Error bars indicate the standard deviation of three replicates. **(B)** Sclerotial formation and colony morphology among wild-type strain, control strain, gene-silenced transformants, and overexpression transformants at 24 h or 15 days after inoculation. WT, Wild-type strain; CK, the control strain.

Cell wall integrity was involved in the formation of sclerotia (Cong et al., 2022), and *SsAGM1* was involved in the maintenance of cell wall integrity and chitin synthesis. This suggested that *SsAGM1* may be involved in vegetative growth, especially the formation of sclerotia. To identify the function of *SsAGM1* in the vegetative growth and formation of sclerotia in *S. sclerotiorum*, WT, CK, gene-silenced transformants, and overexpression transformants were inoculated on PDA plates and cultured at 25°C in the dark. The growth rate of hyphae in gene-silenced transformants was significantly lower than in WT, CK, or overexpression transformants (Figure 6A). WT, CK, and overexpression transformants formed black and mature sclerotia at 15 days after inoculation, but gene-silenced transformants never formed sclerotia even if the culture time was prolonged (Figure 6B). There was no obvious difference in vegetative growth and formation of sclerotia between WT, CK, and overexpression transformants. The results suggested that *SsAGM1* played a critical role in the vegetative growth and formation of sclerotia in *S. sclerotiorum*.

### *SsAGM1* Was Essential for Formation of Infection Cushions in *Sclerotinia sclerotiorum*

**FIGURE 7:**
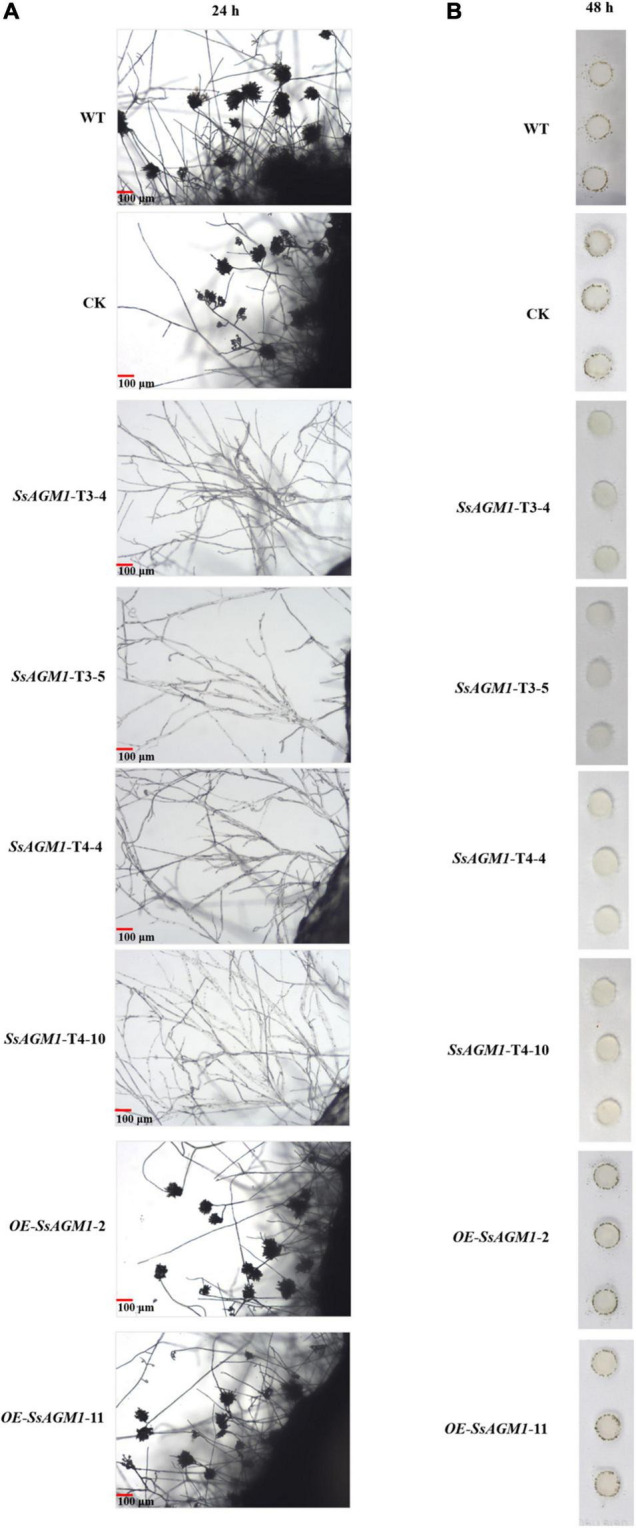
*SsAGM1* is indispensable for the formation of infection cushions in *Sclerotinia sclerotiorum.* **(A)** Micro observation of mycelial morphology on a glass slide at 24 h after inoculation. **(B)** Infection cushions were observed on glass slides that surrounded mycelia-colonized agar plugs at 48 h after inoculation.

The fungal cell wall can sense various stimuli in the external environment, such as hydrophobicity, hardness, plant lipids, plant hormones, or secretory enzymes, and then it can trigger the formation of infection structures during the infection process, such as appressoria or infection cushions (Kou and Naqvi, 2016). Therefore, the disruption of the function and integrity of cell walls usually affects the formation of infected structures. For example, the deletion of *MoTip41* resulted in disruption of CWI, and it resulted in abnormal appressoria in *Magnaporthe oryzae* (Qian et al., 2021). In *Colletotrichum higginsianum*, *ChMK1* played an essential role in cell wall integrity, and the deletion of *ChMK1* resulted in the absence of infection structures (Wei et al., 2016). Formation of infection cushions in WT, CK, gene-silenced transformants, and overexpression transformants was determined on the surface of a hydrophobic interface (glass slide) at 48 h after inoculation. WT and overexpression transformants formed many infection cushions, but the hyphae gathered together and did not form infection structures in gene-silenced transformants (Figures 7A,B). There was no obvious difference in formation of infection cushions between WT, CK, and overexpression transformants. These results suggested that *SsAGM1* was indispensable for formation of infection cushions in *S. sclerotiorum*.

### *SsAGM1* Was Critical for Pathogenicity of *Sclerotinia sclerotiorum*

**FIGURE 8:**
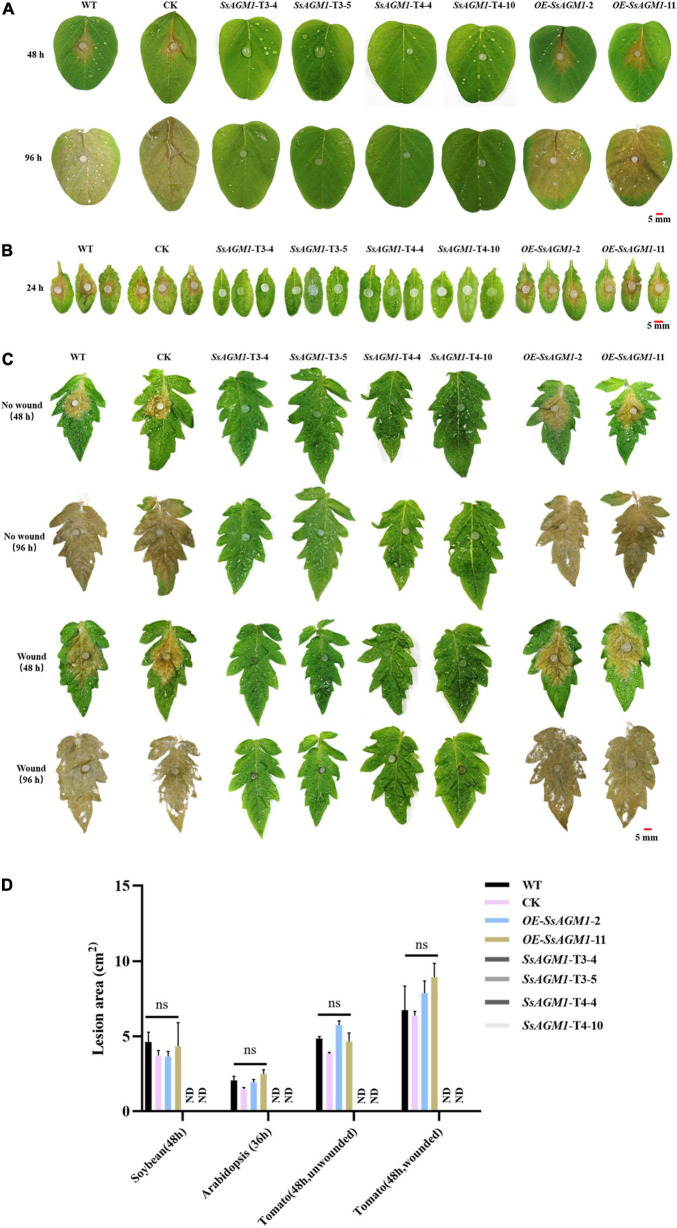
*SsAGM1* is essential for pathogenicity in *Sclerotinia sclerotiorum.* **(A,B)** Disease symptoms of soybean and *Arabidopsis* leaves inoculated with wild-type strain, control strain, gene-silenced transformants, and overexpression transformants. **(C)** Wild-type strain, control strain, gene-silenced transformants, and overexpression transformants were inoculated on intact and wounded tomato leaves. **(D)** Quantitation of lesion sizes in **(A–C)**. The lesion area was calculated by ImageJ. One-way ANOVA; *n* = 3; ns, represents no significant difference from WT at *p* < 0.05. ND, not detected. Data represent means ± SD. WT, Wild-type strain; CK, the control strain.

We investigated the role of *SsAGM1* in *S. sclerotiorum* further for pathogenicity in WT, CK, gene-silenced transformants, and overexpression transformants that were inoculated on intact *Arabidopsis*, tomato, and soybean leaves, and virulence was evaluated 36 or 48 h after inoculation. The WT, CK, and overexpression transformants caused disease symptoms on *Arabidopsis*, tomato, and soybean leaves (Figures 8A–C). By contrast, the gene-silenced transformants were unable to invade intact leaves. Such disease symptoms did not emerge when the incubation time was extended to 4 days (Figures 8A,C), which excluded the possibility that the loss of pathogenicity was attributable to the reduced growth rates of the gene-silenced transformants. The infection experiment was also carried out using wounded tomato leaves, and similar results were obtained (Figure 8C). Inoculated areas at wound sites with gene-silenced transformants did not develop disease symptoms, but this may have caused minor necroses at the margins of the wounds at 4 days after inoculation (Figure 8C). This excluded the possibility of the loss of pathogenicity due to the inability to form infectious structures. In addition, quantitative analysis of the lesion area indicated that the pathogenicity of CK and gene-overexpression transformants were not significantly different from WT (Figure 8D). On the basis of these results, we concluded that *SsAGM1* played an essential role in pathogenicity of *S. sclerotiorum*. However, we need to confirm further the specific mechanism of *SsAGM1* that affected the pathogenicity in *S. sclerotiorum*.

## Discussion

Chitin is one of the most common polysaccharides in nature, which is second only to cellulose in abundance (Brown et al., 2020). As an important component of cell walls, chitin exists in almost all fungal cell walls and plays an important role in the vegetative growth, development, and pathogenicity of pathogenic fungi (Patel and Free, 2019). The biosynthetic pathway of chitin in fungi has been studied in the model organism *S. cerevisiae* (Hofmann et al., 1994; Klis et al., 2006; Milewski et al., 2006), and many enzymes and regulatory factors in this pathway have also been used as candidate targets for fungicides. Chitin is synthesized by UDP-GlcNAc as a precursor (Free, 2013). In the biosynthesis of UDP-GlcNAc, AGM1 catalyzes intramolecular phosphoryl transfer on the phosphosugar substrate GlcNAc-6P, which was a committed step in the synthesis of UDP-GlcNAc (Fang et al., 2013). To date, AGM1 has been functionally studied in only a few species and has not been systemically studied in phytopathogenic fungus. In this study, the *SsAGM1* of *S. sclerotiorum* was identified, and the results indicated that *SsAGM1* plays an important role in the growth, development, cell wall integrity, stress tolerance, and pathogenicity of *S. sclerotiorum*.

*AGM1* was a crucial gene that affected cell survival and the cell cycle in *S. cerevisiae*. After *AGM1* deletion, yeast cannot divide normally, with abnormal morphology and reduced survival rate (Hofmann et al., 1994). In *A. fumigatus*, it failed to construct the *AfAGM1* deletion mutant by knocking out the gene. Finally, inhibiting the expression of *AfAGM1* completely resulted in cell death by constructing the conditional inactivation mutant. The silencing of *AfAGM1* seriously influenced the growth and development in *A. fumigatus* (Fang et al., 2013). In mice, embryos lacking the *AGM1* ortholog (*pgm3*) cannot survive (Greig et al., 2007). These findings suggest that homozygous *SsAGM1* knockout mutants were not screened in this study, possibly because *SsAGM1* is also a lethal gene. We further employed RNAi and overexpression to study the function of *SsAGM1*. To avoid the potentially biased result caused by RNAi technique, four interference targets were designed, and vectors were constructed. Several gene-silenced transformants were obtained from pSD1-SsAGM1-target 3 and pSD1-SsAGM1-target 4 by resistance screening and RT-qPCR analysis (Figure 2). The transformants obtained from the pSilent-Dual1 empty vector was used as CK in this study. In addition, the gene-overexpression vector pYF11:SsAGM1:eGFP was also constructed and gene-overexpression transformants were obtained (Figure 2). Fluorescence microscopy showed that SsAGM1 was located in the cytoplasm of *S. sclerotiorum* (Supplementary Figure 2).

In *A. fumigatus*, the content of UDP-GlcNAc and chitin was significantly reduced in *AfAGM1*-silenced strains (Fang et al., 2013). The hyphae of WT, CK, gene-silenced transformants, and overexpression transformants were stained with CFW. Chitin did not aggregate at the growth point after *SsAGM1* silencing (Figure 3A). The growth point was the most active place for chitin synthesis, and chitin did not gather at the tip of the hyphae, which indicated that chitin synthesis was abnormal. Subsequently, we found by quantitative analysis that the content of UDP-GlcNAc and chitin decreased significantly in *SsAGM1* gene-silenced transformants compared with WT and CK, although the content of UDP-GlcNAc and chitin increased significantly in overexpressed transformants compared with WT (Figures 3B,C). The results showed that *SsAGM1* was required for the synthesis of UDP-GlcNAc and chitin in *S. sclerotiorum*. There are many feedback regulation mechanisms in organisms. Thus, the change in the content of chitin and UDP-GlcNAc may affect the expression level of other genes involved in the UDP-GlcNAc biosynthesis pathway. The decrease in chitin and UDP-GlcNAc content induced the high expression of SS1G-01798, SS1G-14122 (GNA1), and SS1G-05671 (UAP1) in *SsAGM1* gene-silenced transformants. Moreover, the increase in the expression of SS1G-01798 and SS1G-14122 (GNA1) and the decrease in expression of *SsAGM1* resulted in the excessive accumulation of GlcNAc-6P, although the feedback inhibited the expression of SS1G-04070 (GFA1) in *SsAGM1* gene-silenced transformants. However, the expression levels of the four genes were inhibited significantly due to the excessive accumulation of chitin and UDP-GlcNAc in the overexpression transformants (Figure 3D). Staining and quantification assays suggested that *SsAGM1* is involved in the synthesis of chitin in *S. sclerotiorum*.

The fungal cell wall acts as the first physical line of defense against external stress and maintains the shape of cell (Lesage and Bussey, 2006; Geoghegan et al., 2017). Chitin is an important component of cell walls, and is thought to be indispensable for fungal cell wall integrity and rigidity (Patel and Free, 2019). The deletion of chitin synthase results in impaired cell wall integrity has been shown in many important pathogenic fungi, such as *M. oryzae*, *Colletotrichum graminicola*, and *B. cinerea* (Amnuaykanjanasin and Epstein, 2003; Soulié et al., 2003; Kong et al., 2012). Thus, abnormal synthesis of chitin may indicated that cell wall integrity was impaired in *SsAGM1* gene-silenced transformants. In this study, *SsAGM1*gene-silenced transformants were more sensitive to cell wall synthesis inhibitors and osmotic stress compared with WT, whereas the tolerance to cell wall synthesis inhibitors and osmotic stress of *SsAGM1* overexpression transformants increased significantly compared with WT (Figures 4, 5). These results further demonstrated that *SsAGM1* is involved in cell wall integrity and multiple stress tolerance of *S. sclerotiorum*.

Sclerotia are a long-term survival structure of *S. sclerotiorum* and the central component of the infection cycle (Bolton et al., 2006). The development and formation mechanism of sclerotia is an important part of the research in *S. sclerotiorum*. Recent studies have shown that cell wall integrity was related closely to vegetative growth and sclerotia formation of *S. sclerotiorum*. In *S. sclerotiorum*, SsFkh1 is involved in the maintenance of cell wall integrity, and *SsFkh1* deletion mutants are severely defective in hyphal growth and sclerotia formation (Cong et al., 2022). *SsAGM1* was required for chitin synthesis, and it contributed to maintain the integrity of the cell wall. In this study, we found that the silencing of *SsAGM1* significantly inhibited the hyphal growth of *S. sclerotiorum*. In addition, *SsAGM1* gene-silenced transformants never formed sclerotia even if the culture time was prolonged (Figure 6). Our results indicated that *SsAGM1* was essential for vegetative growth and sclerotia formation in *S. sclerotiorum*.

Glycoproteins on the cell wall can sense changes in the external environment and can transmit signals into the cell (Beckerman and Ebbole, 1996; Kou and Naqvi, 2016). In plant pathogenic fungi, hydrophobins on the cell wall provide hydrophobicity to fungal surfaces in contact with air to mediate the attachment of hyphae to the hydrophobic surface, which results in morphogenetic signals (Wosten, 2001). The plant surface is equivalent to a hydrophobic surface, and pathogens recognize the hydrophobic surface, which induced the switching of hyphal morphology (Kou and Naqvi, 2016). In *M. oryzae*, the hydrophobin MPG1 was necessary for host recognition and generated morphogenetic signals that mediated spore germination and differentiation (Beckerman and Ebbole, 1996). Another cell wall-associated proteins, transmembrane mucin Msb2, is one of the key proteins involved in sensing physical stimuli. Deletion of the *MSB2* gene resulted in defects in infection structure and virulence in plant-pathogenic fungi (Lanver et al., 2010; Perez-Nadales and Di Pietro, 2011; Leroch et al., 2015). Thus, the fungal cell wall acts as a signal transduction scaffold for morphogenesis, growth, and virulence, and the disruption of the function and integrity of a cell wall usually affects the formation of infected structures. For example, *MoTip41* was involved in CWI in *M. oryzae*, and the deletion of *MoTip41* resulted in abnormal appressoria (Qian et al., 2021). In *C. higginsianum*, *ChMK1* played an essential role in cell wall integrity, and the *ChMK1* deletion mutant did not form infection structures (Wei et al., 2016). We simulated the hydrophobic surface by using glass slides and observed the infection structure. On glass slides, the hyphae of gene-silenced transformants gathered into bundles, but they could not produce an infection cushion (Figures 7A,B). This indicated that the silencing of *SsAGM1* led to a change in cell wall structure, which affected the receptors on cell walls and the transmission of morphogenetic signals.

Chitin is an important structural compound in both the infection structure and the infection hyphae that are formed in plants. The lack of chitin synthase led to serious pathogenicity defects in several pathogens (Weber et al., 2006; Kim et al., 2009; Larson et al., 2011; Kong et al., 2012; Oliveira-Garcia and Deising, 2013). In plant pathogenic fungi, the delicate regulation of the formation of cell walls was required for structural changes in the cell wall during morphogenesis due to infection (Oliveira-Garcia and Deising, 2013). In general, cell wall integrity is closely related to the virulence of pathogens. In *M. oryzae*, deletion of the chitin synthase-encoding genes *Chs1* and *Chs7* resulted in reduced virulence, and deletion of *Chs6* resulted in loss of pathogenicity (Kong et al., 2012). In *B. cinerea*, chitin synthase mutant (Δ *Bcchs1*) has reduced pathogenicity (Soulié et al., 2003). In *C. graminicola*, the silencing of the β-1,3-glucan synthase gene *GLS1* resulted in severe distortion of necrotrophic hyphae and the loss of pathogenicity (Oliveira-Garcia and Deising, 2013). In this study, we found that the gene-silenced transformants grew on the surface of leaves, but could not invade the inside of the leaf by inoculating the strain on isolated leaves. The gene-silenced transformants could not invade the leaves even at 4 days after inoculation (Figures 8A–C). Myelinolysis was found around the agar block, which may have been caused by the immune response of the host. Furthermore, because the gene-silenced transformants did not form infection cushions, we inoculated strains on the wound of the leaf, but there were still no obvious disease symptoms on the leaf (Figure 8C). This indicated that the silencing of *SsAGM1* caused severe pathogenicity defects in *S. sclerotiorum*, but the pathogenicity defects were not caused mainly by poor growth and the inability to penetrate the host. Therefore, the specific mechanism that allows *SsAGM1* to affect the pathogenicity in *S. sclerotiorum* needs further research.

Collectively, this is the first report on functional characterization of *AGM1* in plant pathogenic fungi. Our results showed that *SsAGM1* was an essential gene for the growth, development, stress response, and pathogenicity in *S. sclerotiorum*. Furthermore, the catalytic mechanism of SsAGM1 also needs to be clarified to develop new fungicide targets.

## Data Availability Statement

The original contributions presented in the study are included in the article/Supplementary Material, further inquiries can be directed to the corresponding author/s.

## Author Contributions

YZ, HP, JZ, and KX planned and designed the research. JZ, HH, and ML performed the experiments. JZ and YZ analyzed the data and wrote the manuscript. HP and YZ provided funding. All authors discussed the data, edited, and approved the manuscript.

## Conflict of Interest

The authors declare that the research was conducted in the absence of any commercial or financial relationships that could be construed as a potential conflict of interest.

## Publisher’s Note

All claims expressed in this article are solely those of the authors and do not necessarily represent those of their affiliated organizations, or those of the publisher, the editors and the reviewers. Any product that may be evaluated in this article, or claim that may be made by its manufacturer, is not guaranteed or endorsed by the publisher.

## Footnotes

1
https://www.ncbi.nlm.nih.gov/
