# Supplementary material for: SsAGM1-Mediated Uridine Diphosphate-N-Acetylglucosamine Synthesis Is Essential for Development, Stress Response, and Pathogenicity of Sclerotinia sclerotiorum
Source: Front Microbiol. 2022 Jun 23;13:938784. doi: 10.3389/fmicb.2022.938784 (PMC9260252; doi:10.3389/fmicb.2022.938784)
Supplement: Supplementary file 1 [file Data_Sheet_1.docx]

Supplementary Material

Supplementary Table 1. Information on the primers used in this study.

| Primers | Sequences 5'-3' |
| --- | --- |
| PSD1-01582-F1 | GCTCTAGACCGTGGTTTGCACTCCTACT |
| PSD1-01582-R1 | GCTCTAGACGGACGAGACGATTGGGTAG |
| PSD1-01582-F2 | GCTCTAGATTGATCCGATGGGCGAGATG |
| PSD1-01582-R2 | GCTCTAGATGAGGCGTCGTAAGCAACTT |
| PSD1-01582-F3 | GCTCTAGACAAAAAGGCTGTTGGGTCGG |
| PSD1-01582-R3 | GCTCTAGAAGTAGGAGTGCAAACCACGG |
| PSD1-01582-F4 | GCTCTAGACGTTGGTCTTGTGGCTGCTC |
| PSD1-01582-R4 | GCTCTAGAACGCTTGTGGTGTTCCCTCC |
| PYF11-01582-F | GTAGGAACCCAATCTTCAAAATGGACGCCAAAATTCTCGAAG |
| PYF11-01582-R | AGCTCCTCGCCCTTGCTCACACCTTTGATGCTTCCCTCAACCC |
| 01582-F1 | CCAAGTCTTTCCCTCTTTCACA |
| 01582-R1 | CTATTCCCTCACTCACAACTACATC |
| PSD1-G418-F | TGTCCGGTGCCCTGAATGAACT |
| PSD1-G418-R | GCCGCCAAGCTCTTCAGCAATAT |
| *SsActin*-F | GTGCCGTTCTCGTCGATTTG |
| *SsActin*-R | TTACCAGCACCGGATTGACC |
| q-01582-F3 | GCTTTGGAAGCTCTTCGTGC |
| q-01582-R3 | CGGACGAGACGATTGGGTAG |
| q-01582-F4 | GGCCACTCTTCTCGACTCAG |
| q-01582-R4 | CATCTCGCCCATCGGATCAA |
| q-01582-F6 | CTACCCAATCGTCTCGTCCG |
| q-01582-R6 | CCACTGGCTCTAGCGAAACT |

Supplementary Table 2. GenBank accession numbers of *Sclerotinia sclerotiorum* and other species in phylogenetic tree.

| Species | GenBank ID |
| --- | --- |
| *Sclerotinia sclerotium* | XP_001597388 |
| *Botrytis cinerea* | XP_024551673 |
| *Aspergillus nidulans* | CBF74428 |
| *Fusarium oxysporum* | XP_018232073 |
| *Neurospora crassa* | XP_963909 |
| *Ustilago maydis* | XP_011391287 |
| *Magnaporthe oryzae* | XP_003713608 |
| *Saccharomyces cerevisiae* | NP_010856 |
| *Schizosaccharomyces pombe* | NP_001342798 |
| *Schizosaccharomyces pombe* | NP_592933 |
| *Candida albicans* | AAF64520 |
| *Penicillium chrysogenum* | KZN92540 |
| *Homo sapiens* | NP_001186848 |
| *Aspergillus fumigatus* | XP_750370 |
| *Drosophila melanogaster* | NP_648588 |
| *Mus musculus* | NP_082628 |
| *Danio rerio* | NP_001007054 |
| *Fusarium verticillioides* | XP_018743957 |
| *Verticillium dahliae* | XP_009655356 |

Supplementary Table 3. The content of UDP-GlcNAc and Chitin.

|  | UDP-GlcNAc (ng/g) | | | Chitin [μg GlcNAc/5 mg (hyphae dry weight)] | | |
| --- | --- | --- | --- | --- | --- | --- |
| WT | 33.78 | 33.69 | 34.05 | 7.59 | 8.47 | 8.25 |
| CK | 34.11 | 32.51 | 32.70 | 7.60 | 7.58 | 7.76 |
| *SsAGM1*-T3-4 | 29.14 | 30.76 | 29.04 | 5.83 | 7.59 | 6.49 |
| *SsAGM1*-T3-5 | 29.60 | 31.10 | 29.13 | 6.41 | 6.85 | 6.71 |
| *SsAGM1*-T4-4 | 29.50 | 30.00 | 29.30 | 5.18 | 5.39 | 6.05 |
| *SsAGM1*-T4-10 | 30.10 | 29.60 | 29.81 | 5.39 | 6.05 | 6.71 |
| *OE- SsAGM1*-2 | 36.12 | 34.77 | 35.22 | 8.39 | 10.12 | 9.49 |
| *OE- SsAGM1*-11 | 38.47 | 37.30 | 36.21 | 8.67 | 8.82 | 9.78 |


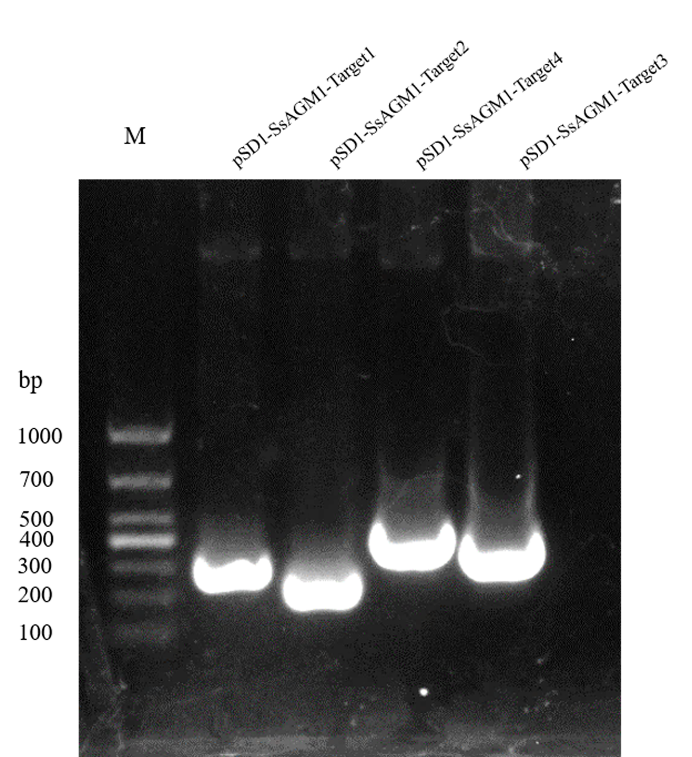


**Supplementary Figure 1.** Identifification of *SsAGM1* gene-silencing vector by PCR. M, DL1000, DNA marker; lanes 2–5: pSD-SsAGM1-T1, pSD-SsAGM1-T2, pSD-SsAGM1-T1and pSD-SsAGM1-T4 vector.


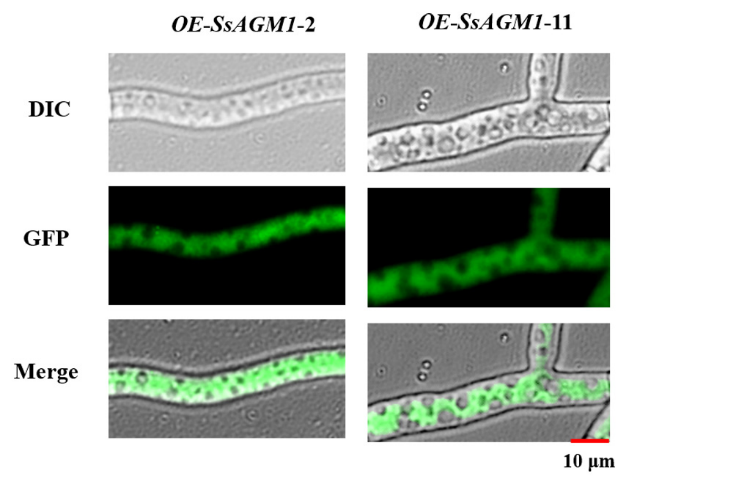


**Supplementary Figure 2.** SsAGM1 localizes to the cytoplasm in *Sclerotinia sclerotiorum*. SsAGM1-GFP is strongly expressed in cytoplasm. Scale bars, 10 μm. Gene-overexpression transformants were inoculated on PAD covering cellophane and observed at 12 h after inoculation.
